# Zika virus antagonizes interferon response in patients and disrupts RIG-I–MAVS interaction through its CARD-TM domains

**DOI:** 10.1186/s13578-019-0308-9

**Published:** 2019-06-07

**Authors:** Yiwen Hu, Xinhuai Dong, Zhenjian He, Yun Wu, Shihao Zhang, Jiajie Lin, Yi Yang, Jiahui Chen, Shu An, Yingxian Yin, Zhiyong Shen, Gucheng Zeng, Han Tian, Junchao Cai, Yi Yang, Hongyu Guan, Jueheng Wu, Mengfeng Li, Xun Zhu

**Affiliations:** 1 Key Laboratory of Tropical Disease Control (Sun Yat-sen University), Ministry of Education, Guangzhou, 510080 China; 2 Changsha Customs District P. R. China, Changsha, 410000 China; 3 Department of Microbiology, Zhongshan School of Medicine, Sun Yat-sen University, Guangzhou, 510080 China; 4 School of Public Health, Sun Yat-sen University, Guangzhou, 510080 China; 5 Department of Basic Medicine, Zhongshan School of Medicine, Sun Yat-sen University, Guangzhou, 510080 China; 6 Department of Clinical Medicine, Zhongshan School of Medicine, Sun Yat-sen University, Guangzhou, 510080 China; 7 Guangzhou Institute of Pediatrics, Guangzhou Women and Children’s Medical Center, Guangzhou Medical University, Guangzhou, 510623 China; 8 Department of Pharmacology, Zhongshan School of Medicine, SunYat-sen University, Guangzhou, 510080 China; 9 Department of Endocrinology, The First Affiliated Hospital of SunYat-sen University, Guangzhou, 510080 China; 10 Guangdong Provincial Key Laboratory of Orthopedics and Traumatology, Guangzhou, 510080 China

**Keywords:** Zika virus, Nonstructural protein 4A, Interferon, RIG-I, MAVS

## Abstract

**Background:**

The emerging threat to global health associated with the Zika virus (ZIKV) epidemics and its link to severe complications highlights a growing need to better understand the pathogenic mechanisms of ZIKV. Accumulating evidence for a critical role of type I interferon (IFN-I) in protecting hosts from ZIKV infection lies in the findings that ZIKV has evolved various strategies to subvert the host defense line by counteracting the early IFN induction or subsequent IFN signaling. Yet, mechanisms underlying the counter-IFN capability of ZIKV and its proteins, which might contribute to the well-recognized broad cellular tropisms and persistence of ZIKV, remain incompletely understood.

**Results:**

Using RNA sequencing-based transcriptional profiling of whole blood cells isolated from patients acutely infected by ZIKV, we found that transcriptional signature programs of antiviral interferon-stimulated genes and innate immune sensors in ZIKV-infected patients remained inactive as compared to those of healthy donors, suggesting that ZIKV was able to suppress the induction of IFN-I during the natural infection process in humans. Furthermore, by analyzing the molecular interaction in a ZIKV NS4A-overexpression system, or in the context of actual ZIKV infection, we identified that ZIKV NS4A directly bound MAVS and thereby interrupted the RIG-I/MAVS interaction through the CARD-TM domains, leading to attenuated production of IFN-I.

**Conclusions:**

Our findings collectively revealed that ZIKV NS4A targeted MAVS and contributed to ZIKV immune evasion through abrogating MAVS-mediated IFN production. These findings obtained from patient studies have added new knowledge and molecular details to our understanding regarding how ZIKV mediates suppression of the IFN-I system and may provide a new basis for the future development of anti-ZIKV strategies.

**Electronic supplementary material:**

The online version of this article (10.1186/s13578-019-0308-9) contains supplementary material, which is available to authorized users.

## Background

Zika virus (ZIKV) is an emerging mosquito-borne pathogen belonging to the genus *Flavivirus* of the family *Flaviviridae*, which also includes several life-threatening human pathogens, such as Japanese encephalitis virus (JEV), dengue virus (DENV), yellow fever virus (YFV) and West Nile virus (WNV) [1]. While ZIKV was originally identified in the Zika forest of Uganda in 1947 and the first human infection case was documented in 1954, the most serious Zika pandemic to date began in the Americas in 2013–2014 [1, 2]. As of December 2018, over 86 countries and territories have reported cases of active ZIKV transmission [3]. Clinically, although most Zika cases are asymptomatic or only manifest as an influenza-like illness, severe forms of ZIKV infection such as microcephaly and other neurological abnormalities in newborns and Guillain–Barré syndrome, meningoencephalitis, multi-organ failure or thrombocytopenia in adults are seen in the clinic [1, 4]. Thus far, no clinically approved vaccines or specific anti-ZIKV drugs are available for the control of the disease [1]. Hence, understanding the molecular basis and host immune mechanisms based on which severe diseases develop as a result of ZIKV infection is key to developing strategies against ZIKV-associated conditions.

While it is beginning to be revealed that mutations in the prM [5] and NS1 [6] proteins might enable ZIKV to become more virulent and/or transmissible, mounting evidence suggests that innate immunity is involved in the governance of ZIKV replication and pathogenesis [1, 7]. The interferon (IFN) system, being an essential component of innate immunity, constitutes the first line of defense against viral infection, including ZIKV [7]. A vital role for the IFN system in abrogating ZIKV infection in the host was revealed by the fact that ZIKV is pathogenic in IFN receptor knockout mice but not in immunocompetent mice [8–10]. Moreover, Smith et al. demonstrated the neuropathogenesis of ZIKV in a highly susceptible immunocompetent mouse model after the function of type I interferon was blocked by an antibody [11]. These findings, together with several other lines of evidence [12–14], support a notion that in order for a pathogenic infection to be established, ZIKV needs to attenuate the production of IFNs or the anti-viral effects of the IFN system.

As is widely acknowledged, activation of IFN signaling is initiated by recognition of viral pathogen-associated molecular patterns (PAMPs) through the pathogen recognition receptors (PRRs) [1, 7]. RIG-I-like receptors (RLRs) and cGMP-AMP synthase (cGAS) have been identified as two of the most important PRRs for the detection of ZIKV infection [7, 15]. RIG-I recognizes cytosolic viral RNA of ZIKV, which triggers a RIG-I conformational change to expose the caspase activation and recruitment domains (CARD), subsequently triggering the downstream signaling cascades by interacting with the N-terminal CARD-containing mitochondrial antiviral signaling (MAVS) adaptor protein, resulting in the activation of IFN regulatory factor 3 (IRF3) via TANK-binding kinase 1 (TBK1) and IκB kinase ε (IKKε) [7, 16]. cGAS is mainly known as a critical sensor for DNA viruses, and yet recent findings indicate that the cGAS-STING pathway might also be involved in responding to infection of positive-sense, single-stranded RNA viruses, including ZIKV [17]. STING has also been found to interact with MAVS, most likely at the mitochondrion or mitochondria-associated membranes (MAMs), which is followed by recruitment of TBK1 and IRF3, leading to the production of type I IFN [17].

It is well established that viruses have co-evolved with their hosts, accompanied by development of multiple strategies to evade and antagonize the host IFN system [7]. Previous studies have shown that most flaviviruses have evolved diverse strategies to abrogate the induction of IFN and the downstream signaling pathways. Currently available data suggest that ZIKV infection can inhibit type I IFN responses in dendritic cells (DCs) through downregulation of IFN-stimulated genes and impair DC function [18], yet the interferon response in other cell types remains unknown. ZIKV genome codes for a single polyprotein that is post-translationally, proteolytically processed by viral and host proteases to produce ten proteins, including three structural proteins (capsid, membrane precursor, and envelope), and seven nonstructural proteins (NS1, NS2A, NS2B, NS3, NS4A, NS4B, and NS5) [1]. The ZIKV nonstructural proteins NS1, NS2B3, NS4B, and NS5 have been implicated to be engaged in ZIKV immuno pathogenicity through diverse strategies. For example, NS1 and NS4B of ZIKV inhibit RLR-induced IFN-β production via interacting with TBK1 and thereby blocking TBK1 oligomerization [6, 19]. NS2B3 impairs the IFN signaling pathway by facilitating proteasomal degradation of Jak1 [19] and STING [17]. Furthermore, ZIKV NS5 has been found to bind and degrade host STAT2 and consequently block the IFN response [20, 21]. It remains to be determined whether other viral factors also contribute to the evasion of the innate immune response by ZIKV.

Since MAVS signaling is essential for inducing an IFN production during ZIKV infection, it is plausible that viruses may hijack this important host response to facilitate infection of the host. Our previous findings revealed that DENV NS4A protein played an important role in suppressing interferon production through binding MAVS and disrupting the RIG-I–MAVS interaction [22]. Based on the possible functional similarities but mechanistic diversities by which the NS4A of the well-known flaviviruses DENV1-4 inhibits IFN signaling, we asked whether this paradigm applies to another member of the flavivirus group, ZIKV. This current study found that ZIKV interfered with the RIG-I–MAVS signaling to attenuate type I IFN production. More specifically, our results suggest that ZIKV NS4A targets MAVS and interrupts the RLR–MAVS interaction, leading to reduced induction of type I IFNs.

## Results

### ZIKV suppresses type I interferon production in human subjects and in host cells

**Fig. 1:**
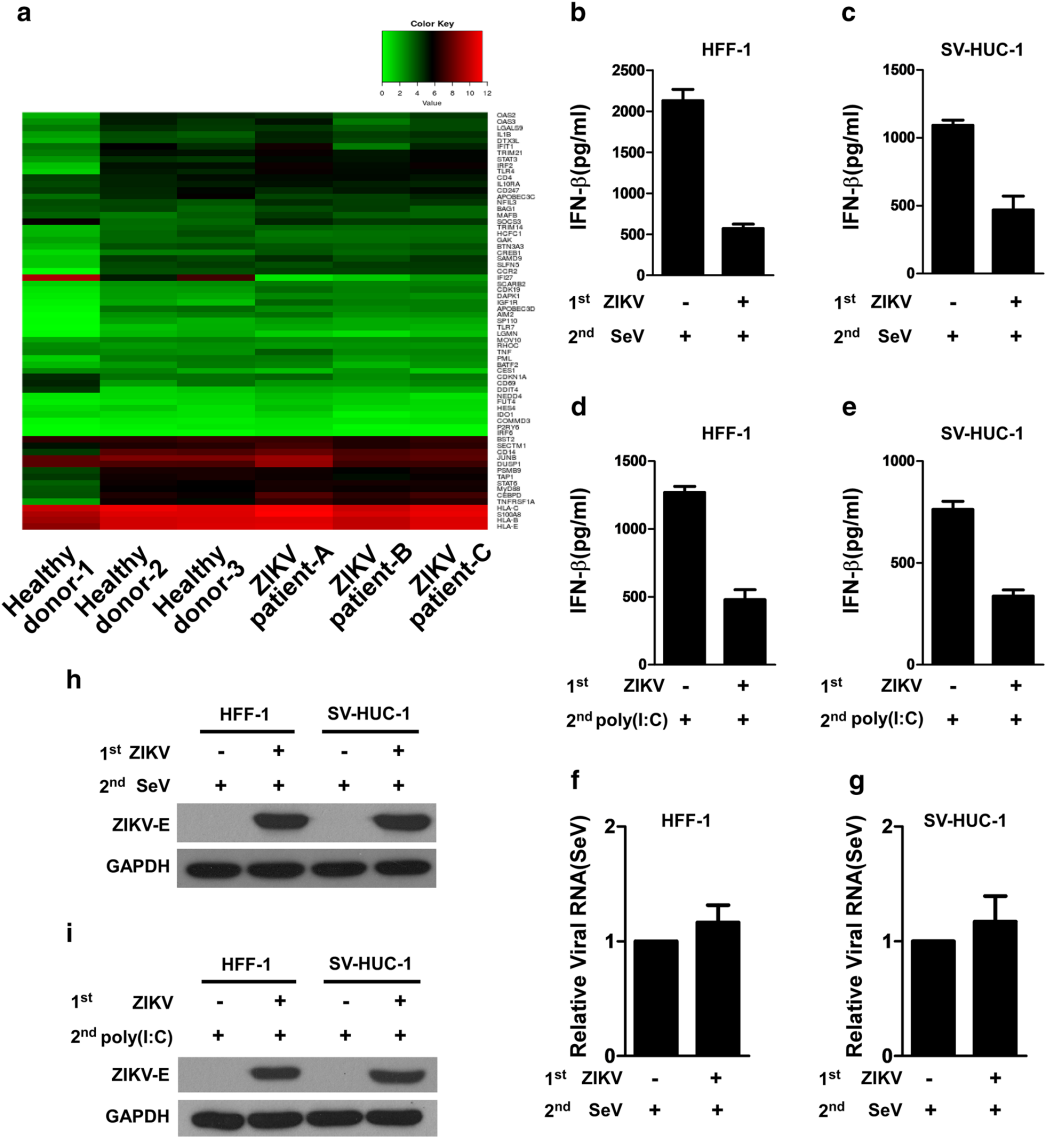
ZIKV abrogates the activation of type I IFN signaling. **a** Heatmap of a pre-defined gene list of type I IFN and ISGs that are differentially expressed (DEGs), generated from comparative RNA-seq data among three ZIKV-infected patients (ZIKV patients **a**–**c**) and three healthy donors. The expression of read-mapped genes normalized across the entire dataset was analyzed based on the RPKM value, and *p*-values < 0.05 and log_2_ fold change ≥ 1.5 relative to the control cohort were set as the threshold to assess the statistical significance of the differential gene expression. **b**–**e** Infection with ZIKV reduces type I IFN production after a secondary infection. HFF-1 and SV-HUC-1 cells were infected with mock or ZIKV at an MOI of 1, and 24 h later, respectively, infected with SeV at 100 HAU/ml (**b**, **c**) or transfected with 20 μg/ml of poly(I:C) (**d**, **e**). IFN-β protein levels were measured using specific ELISA in the supernatants collected at 36 h after secondary-infection from the two cell lines, and cell lysates were harvested for real time RT-PCR to determine the RNA level of SeV (**f**, **g**), and for Western blotting analysis to determine the levels of ZIKV envelope protein (E) and GAPDH protein (**h, i**). Data are shown as the mean ± SD derived from three repeatedexperiments. *p < 0.05, and **indicates *p *< 0.01 (Student’s *t* test)

To assess whether ZIKV infection influences the production of type I IFN in patients, we collected whole blood cells from ZIKV-infected human subjects and conducted RNA sequencing (RNA-seq)-based transcriptional profiling experiments to characterize the expression of type I IFN genes and their downstream ISGs. As shown in Fig. 1a, whole blood cells were obtained from three ZIKV-infected patients, whose clinical features were reported in our previous study [23]. The clinical characteristics of three individuals with ZIKV infection and the background of three healthy donors are summarized and compared in Additional file 1: Table S1. Upon analyzing the global transcriptional profiling data obtained from the whole blood cells of the ZIKV-infected individuals, we found that in consistence with other observations, 92.75% of the genes listed in a pre-defined genes list of type I IFN and ISGs (69 genes) with known antiviral activity against flavivirus [18, 24] showed no or little change when comparing ZIKV-infected patients and uninfected controls (Fig. 1a and Additional file 2: Table S2), with only two genes (NFIL3 and IGF1R) down-regulated and one (IFI27) up-regulated (*p *< 0.05), and two undetected (TREX1 and DCP1A). To further investigate the ability of ZIKV to block IFN production in response to general viral attack in cultured human cells, we infected HFF-1 and SV-HUC-1 cells with ZIKV and then used SeV or poly(I:C) to secondarily infect the cells. SeV or poly(I:C) was used in this experiment as it represents a well-recognized potent inducer of type I IFN. ELISA analysis showed that while SeV or poly(I:C) treatment alone induced drastic production of IFN-β, pre-infection of the cells with ZIKV robustly lowered the level of IFN-β produced in response to secondary SeV infection or poly(I:C) treatment (Fig. 1b–e). SeV infection in HFF-1 and SV-HUC-1 was validated by real time RT-PCR to detect the SeV RNA genome (Fig. 1f, g), which clearly shows the same RNA expression level of SeV in each group. ZIKV infection in HFF-1 and SV-HUC-1 was validated by western blotting analysis to detect the ZIKV envelope protein (E) (Fig. 1h, i). These data demonstrated that ZIKV could actively suppress the type-I IFN response in host cells and patients, warranting further investigation of how ZIKV suppresses IFN production in human host cells.

### ZIKV NS4A protein abrogates IFN-I signaling

**Fig. 2:**
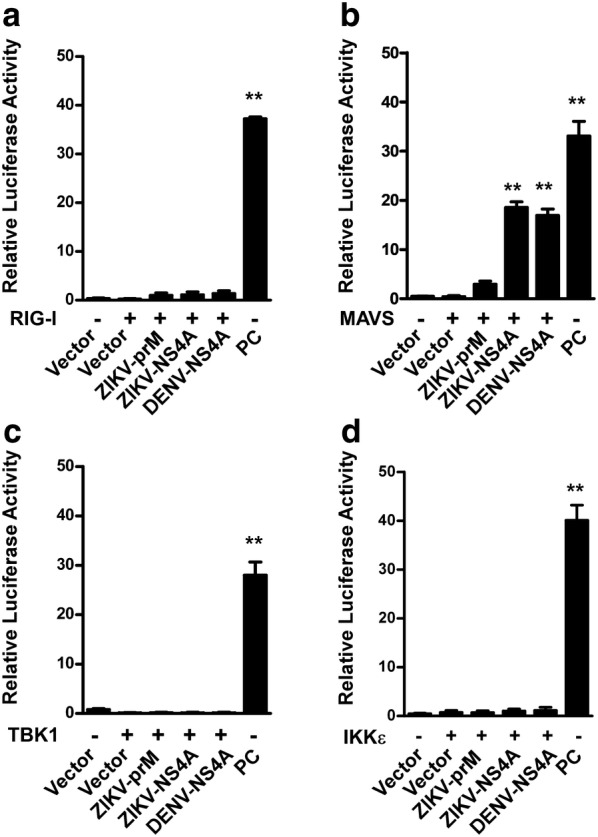
Molecular interaction between ZIKV NS4A protein and the type I IFN induction pathway. GAL4-based mammalian two-hybrid screening assays were performed to identify the molecular targets of ZIKV proteins in RIG-I signaling. 293T cells in 24-well plates were co-transfected with a pGL4.31 vector, a pFN11A (BIND) vector expressing a fusion protein of GAL4-BD and individual ZIKV prM, NS4A or DENV NS4A proteins, and a pFN10A (ACT) vector expressing **a** RIG-I, **b** MAVS, **c** TBK1 or **d** IKKε. The pFN11A (BIND) vector contained a *Renilla* luciferase gene that was used as an internal control to normalize DNA transfection efficiency. The pBIND and pACT vectors were used as negative controls, and the pBIND-Id and pACT-MyoD vectors were used as positive controls (PCs) according to the manufacturer’s instructions. At 48 h post-transfection, cell lysates were harvested for the luciferase activity assay. The results are shown as relative luciferase activity after normalization with *Renilla* luciferase activity. Data are shown as the mean ± SD derived from three repeat experiments. *p < 0.05, and **p < 0.01 (Student’s t test)

Our previous finding that the NS4A protein of all four serotypes of DENV, which also belongs to the genus *Flaviviridae*, blocks type I IFN production through targeting RIG-I–MAVS-mediated signaling [22] prompted us to ask whether ZIKV NS4A protein is also able to interfere with RLR–MAVS-dependent IFN production. To this end, mammalian two-hybrid analysis was performed to identify possible molecular interactions between ZIKV NS4A and components along the RIG-I signaling pathway. Specifically, we used ZIKV NS4A to transfect 293T cells, together with, respectively, one of the four key RLR signaling proteins, namely, RIG-I, MAVS, TBK1 and IKKε, whose expression was verified by Western blotting (Additional file 3: Figure S1, A–D). When DENV NS4A and ZIKV prM were used as a positive and negative control, respectively, for NS4A binding, our results showed a strong interaction between MAVS and ZIKV-NS4A, DENV2-NS4A (Fig. 2b), whereas no interaction between ZIKV prM, NS4A and RIG-I, TBK1 orIKKε was found (Fig. 2a, c, d), suggesting that MAVS might represent a specific interaction partner of ZIKV NS4A.

**Fig. 3:**
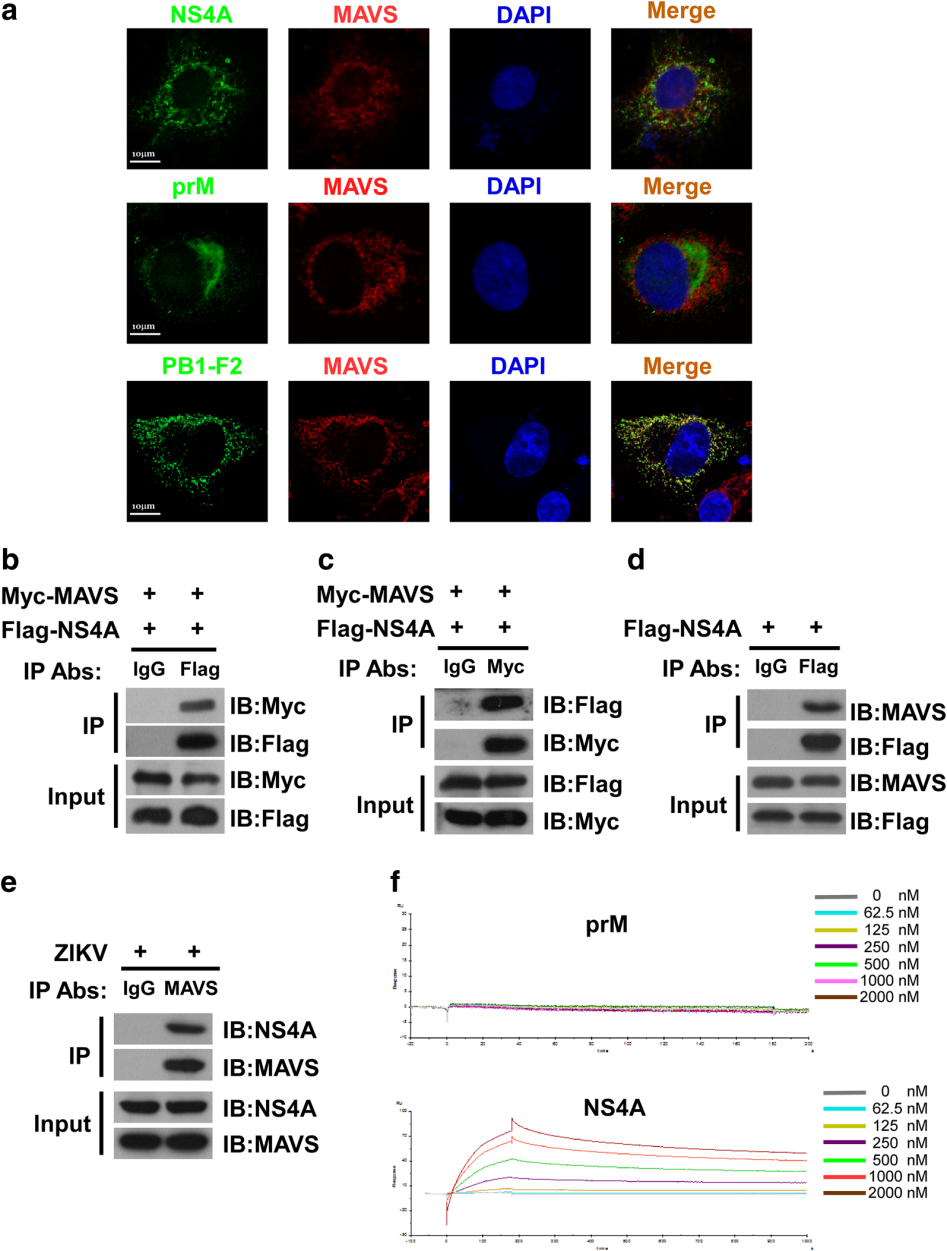
ZIKV NS4A co-localizes and interacts with MAVS. **a** HeLa cells transfected with plasmids expressing Flag-tagged NS4A, Flag-tagged ZIKV prM or influenza Flag-tagged PB1-F2 were stained with anti-Flag and anti-MAVS antibodies as well as DAPI. Secondary antibodies conjugated to rhodamine and FITC dye were used to visualize the indicated proteins. Images are representative of three independent experiments. 293T cells co-transfected with plasmids encoding Myc-tagged MAVS and Flag-tagged NS4A were used in a co-IP assay to address whether ZIKV NS4A protein physically interacts with MAVS. Cell lysates were precipitated with an anti-Flag antibody (**b**), anti-Myc antibody (**c**), or control mouse IgG, and immunocomplexes were analyzed with the indicated antibodies by western blotting. **d** 293T cells were transfected with plasmids encoding Flag-tagged NS4A, followed by immunoprecipitation using anti-Flag antibody or control IgG. The immunocomplexes were analyzed with anti-MAVS antibody by Western blotting. **e** HFF-1 cells were infected with ZIKV at an MOI of 5 followed by immunoprecipitation using anti-MAVS antibody or control mouse IgG. The immunocomplexes that were captured by the protein G Dynabeads were analyzed by Western blotting using anti-NS4A, or anti-MAVS antibodies. **f** SPR analysis of the interactions between MAVS and NS4A. Direct binding was measured by Biacore assays. MAVS was immobilized on a CM5 chip. The analytes consisted of serial dilutions of NS4A proteins ranging between 0 and 2000 nM. The data shown are representative of three independent experiments with similar results

To verify the above finding that ZIKV NS4A interacts with MAVS, we examined whether NS4A of Zika virus co-localizes with MAVS by performing confocal microscopy in cells expressing Flag-tagged NS4A. Our results revealed that, as shown in Fig. 3a, when influenza (IVA)PB1-F2, a viral protein well recognized for its strong ability to antagonize type I IFN induction by targeting MAVS, was employed as a positive control, ZIKV NS4A or PB1-F2, respectively, but not ZIKV prM, was found to co-localize with MAVS, supporting the notion that ZIKV NS4A interacts with MAVS in host cells.

Furthermore, a co-immunoprecipitation (co-IP) assay was performed to reveal a physical interaction between ZIKV NS4A and MAVS. 293T cells were co-transfected with Myc-tagged MAVS and Flag-tagged NS4A, and cell lysates were precipitated with anti-Flag antibody or control mouse IgG. Our results showed that Myc-tagged MAVS was detected in the precipitate pulled down by the anti-Flag antibody (Fig. 3b). Reverse immunoprecipitation, using an anti-Myc antibody to precipitate ectopic NS4A followed by using an anti-Flag antibody for western blotting analysis also confirmed that NS4A and MAVS were present in the immunocomplexes (Fig. 3c). To include a vector control, 293T cells were co-transfected with Myc-tagged MAVS and pcDNA3.1 vector, and cell lysates were precipitated with anti-Myc antibody or control mouse IgG. Furthermore, 293T cells were co-transfected with Flag-tagged NS4A and pcDNA3.1 vector, and cell lysates were precipitated with anti-Flag antibody or control mouse IgG. The results showed that there were not specific bands present in the immunoprecipitates pulled down by the anti-Flag or anti-Myc antibody in the vector control group (Additional file 3: Figure S2). An IP assay was also performed to precipitate endogenous MAVS using Flag-tagged NS4A. Our results showed that endogenous MAVS could be pulled down together with Flag-tagged NS4A (Fig. 3d). To further elucidate the physical interaction between NS4A and MAVS, we also performed an endogenous IP assay upon ZIKV infection. When HFF-1 cells were infected with ZIKV and the cell lysates were subsequently precipitated using an antibody against MAVS to pull down its interacting proteins, our results revealed that an antibody against MAVS could pull down ZIKV NS4A (Fig. 3e), suggesting that NS4A and MAVS were complexed in ZIKV-infected cells.

The binding affinity of NS4A and MAVS were then analyzed by the Biacore surface plasmon resonance technology. In brief, the reference surface of the CM5 sensor chip was immobilized with MAVS, followed by injection of various concentrations of purified recombinant NS4A protein, or prM, or blank control sample for response measurement. As shown in Additional file 3: Figure S3, our data showed no binding affinity between blank control sample and MAVS protein. Furthermore, as shown in Fig. 3f, a significantly increased binding affinity between NS4A and MAVS was demonstrated, strongly suggesting that NS4A directly bound MAVS.

### ZIKV NS4A interacts with both the CL and TM domains of MAVS and prevents RIG-I from complexing with MAVS

**Fig. 4:**
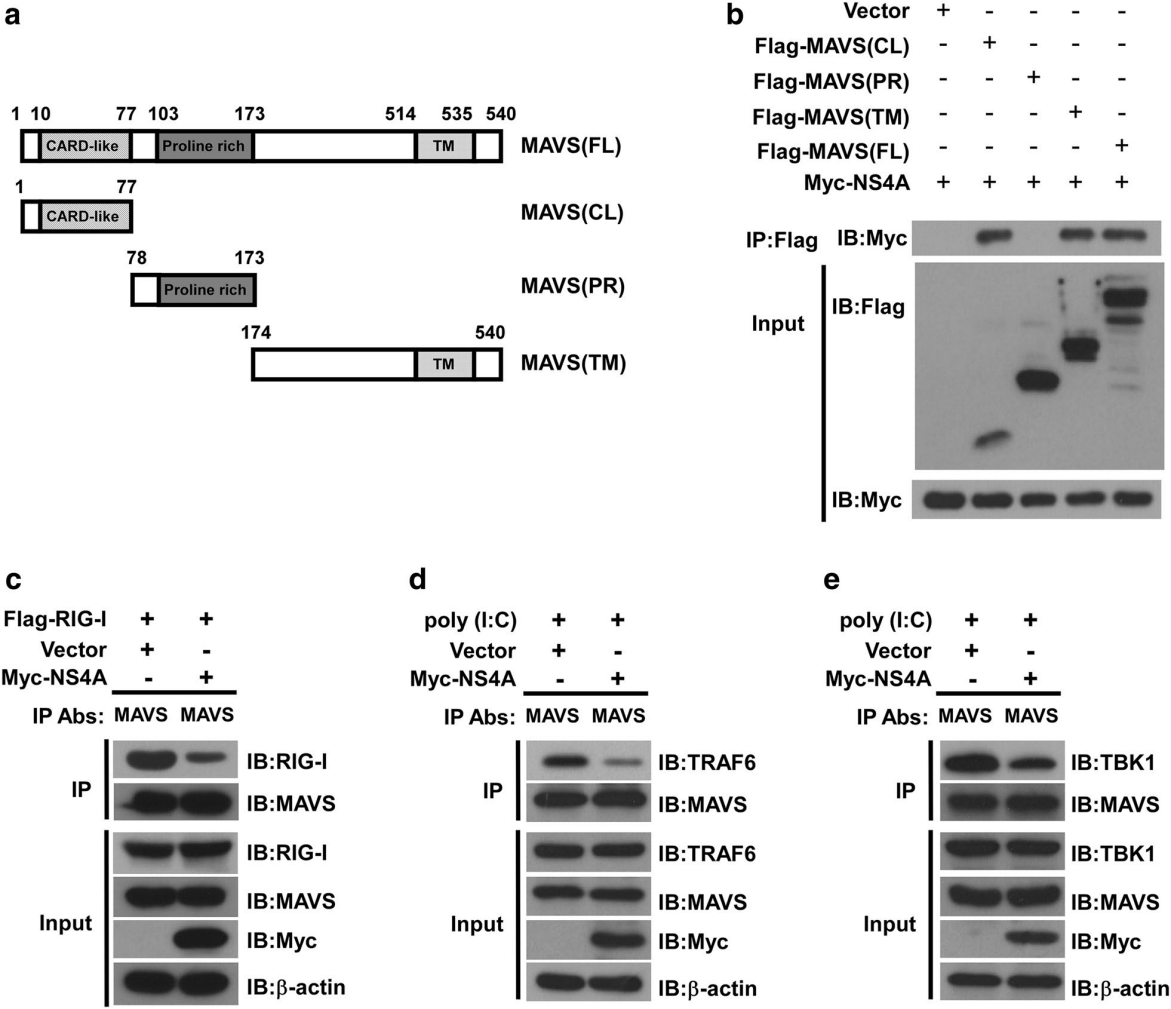
ZIKV NS4A interacts with both the CL and TM domains of MAVS and prevents RIG-I from binding MAVS. **a** A schematic diagram of the MAVS protein and functional domains: CARD-like domain (aa 10 to 77), proline-rich domain (aa 103 to 173) and transmembrane domain (aa 514 to 535). **b** Co-IP and western blotting analysis of 293T cells transfected with Myc-tagged NS4A along with vectors expressing the indicated Flag-tagged MAVS truncation forms or full-length MAVS. Empty vector was used as a negative control. **c** 293T cells were co-transfected with Flag-tagged RIG-I and Myc-tagged NS4A or an empty vector control for 24 h. Whole cell lysates were subjected to immunoprecipitation using an anti-MAVS antibody and analyzed by western blotting for RIG-I (**c**). 293T cells were transfected with Myc-tagged NS4A or an empty vector control for 24 h later, transfected with 20 μg/ml of poly(I:C). Whole cell lysates were subjected to immunoprecipitation using an anti-MAVS antibody and analyzed by western blotting for TRAF6 (**d**), or TBK1 (**e**). The expression of precipitated proteins was determined by western blotting analysis using the indicated antibodies (lower panel). The data shown are representative of three independent experiments with similar results

As MAVS is a 540-amino acid (aa) protein consisting of three structural and functional domains, including an N-terminal CARD-like (CL) domain, a mid-region proline-rich (PR) domain and a C-terminal transmembrane (TM) domain [16], to map the NS4A-binding domain(s) in MAVS, we generated serial MAVS deletion mutants, i.e., a Flag-MAVS (CL) construct expressing aa 1–77 containing the CARD-like domain, a Flag-MAVS (PR) construct expressing aa 78–173 containing the proline-rich domain, a Flag-MAVS (TM) construct expressing aa 174–540 containing the transmembrane domain, and a Flag-MAVS (FL) construct expressing full-length MAVS (Fig. 4a). 293T cells were transfected with Myc-tagged NS4A together with each of the above Flag-tagged MAVS plasmids or empty control vector, respectively. Our co-IP assay showed that either the CL or TM domain of MAVS could pull down NS4A (Fig. 4b), suggesting a key role for the CL and TM domains in the interaction between NS4A and MAVS.

Notably, previous studies suggested that MAVS transduced signals from RIG-I through their CARD–CARD domain interaction, leading to activation of IRF3 and IFN induction [15]. Interestingly, our current data also showed that NS4A interacted with the CARD-like domain of MAVS, thereby prompting us to investigate whether NS4A affects the interaction between RIG-I and MAVS. To address this question, we examined the formation of the RIG-I–MAVS complex in the presence or absence, respectively, of NS4A. 293T cells were transfected with Flag-tagged RIG-I together with Myc-tagged NS4A or control vector. The cell lysates were immunoprecipitated with anti-MAVS antibody and analyzed by Western blotting with anti-MAVS antibody or anti-RIG-1 antibody. We found that RIG-I strongly interacted with MAVS in the absence of NS4A, whereas cells expressing NS4A exhibited a significant decrease in the interaction between RIG-I and MAVS (Fig. 4c). Furthermore, when using poly(I:C) as a stimulus for MAVS signaling, we performed an immunoprecipitation assay by using an antibody against MAVS for the pull-down of TRAF6 or TBK1 proteins and an antibody against TRAF6 or TBK1 for the blotting detection. We found that overexpression of NS4A exhibited a significant decrease in the interaction between MAVS and its downstream effectors TRAF6 or TBK1 (Fig. 4d, e). In summary, these data suggested that NS4A bound MAVS, which subsequently prevented its interaction with RIG-I, modulated the downstream signaling, and consequently suppressed IFN production.

### ZIKV NS4A-MAVS interaction inhibits IFN induction and viral infection

**Fig. 5:**
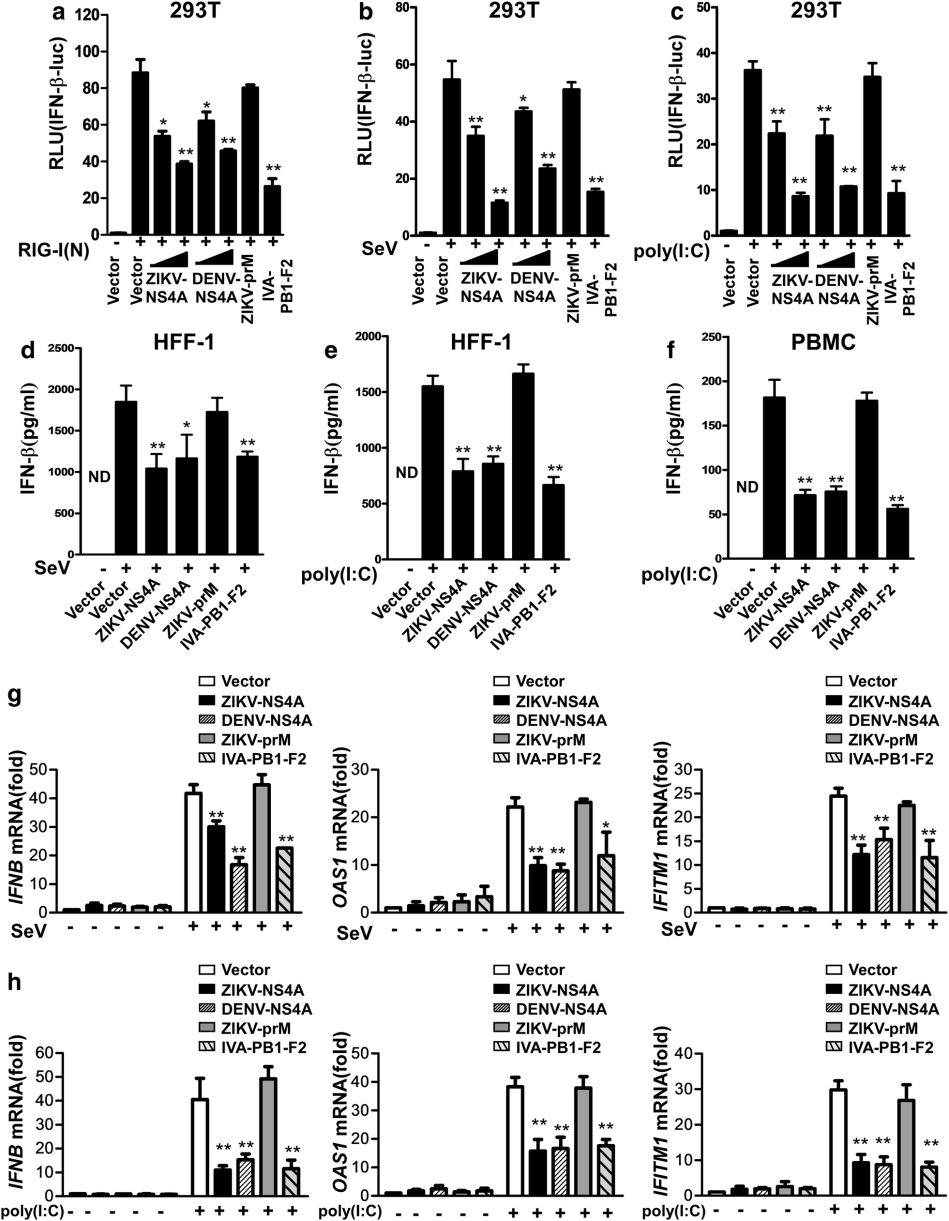
ZIKV NS4A negatively regulates IFN production. **a** Lysates of 293T cells co-transfected with RIG-I(N), IFN-β reporter, together with ZIKV NS4A (250, or 500 ng), DENV NS4A (250, or 500 ng), ZIKV prM (500 ng) or PB1-F2 (500 ng)were analyzed for luciferase activity. 293T cells were transfected with IFN-β reporter, together with ZIKV NS4A (250, or 500 ng), DENV NS4A (250, or 500 ng), ZIKV prM (500 ng) or PB1-F2 (500 ng), **b** then infected with SeV at 100 HAU/ml for 24 h, **c** or transfected with 20 μg/ml of poly(I:C) for 24 h followed by analysis of the cell lysates for luciferase activity. **d**, **e** HFF-1 cells or **f** PBMC were transfected with blank control vector or a vector expressing ZIKV NS4A (500 ng), DENV NS4A (500 ng), ZIKV prM (500 ng) or PB1-F2 (500 ng), respectively, and 24 h later were mock-infected or infected with SeV at 100 HAU/ml or transfected with or without 20 μg/ml of poly(I:C) for 24 h, followed by analysis of the supernatant for IFN-β protein levels. **g**, **h** Meanwhile, at 24 h post-infection, total cellular RNA was isolated, and real-time PCR was performed to detect *IFNB*, *OAS1*, and *IFITM1* mRNA levels in 293T cells. The data are presented as the mean ± SD derived from three repeat experiments. *p < 0.05, and **p < 0.01 (Student’s t-test)

As the aforementioned data showed that ZIKV NS4A interacts with MAVS, we next sought to determine whether such an interaction effects on type I IFN production. We co-transfected 293T cells with RIG-I(N), which is well known to stimulate IFN-β production, together with increasing amounts of the expression vector containing ZIKV NS4A plus the IFN-β luciferase reporter cassette. Our results demonstrated that when ZIKV prM was used as a negative control and the PB1-F2 protein of influenza virus and DENV NS4A were used as a positive control, overexpression of ZIKV NS4A indeed inhibited the reporter luciferase activity driven by RIG-I(N) (Fig. 5a and Additional file 3: Figure S4 A–C). Furthermore, PB1-F2 or DENV NS4A, but not prM, significantly decreased the IFN-β reporter luciferase activity driven by RIG-I(N) (Fig. 5a), indicating that NS4A was capable of interfering with the promoter activity of IFN-β.

To further examine the effect of ZIKV NS4A on IFN-β production induced by viral infection, we used SeV or poly(I:C) as an IFN stimulus and found that expression of ZIKV NS4A, DENV NS4A or influenza PB1-F2, but not ZIKV prM, markedly inhibited the activities of IFN-β reporters upon induction by SeV (Fig. 5b) or poly(I:C)(Fig. 5c). Furthermore, ELISA analysis was applied to measure the expression level of IFN-I produced in infected cells, and we found that ectopic expression of ZIKV NS4A reduced IFN-β production stimulated by SeV (Fig. 5d)or poly(I:C) (Fig. 5e) in 293T cells, and also in primary PBMC stimulated by poly(I:C) (Fig. 5f). Taken together, these data indicated that ZIKV can efficiently blocked the type-I IFN response, and that NS4A blocked the RNA-level expression of endogenous human *IFNB* and ISGs (Fig. 5g, h), suggesting a pivotal role for ZIKV NS4A in suppressing type I IFN signaling via interacting with MAVS.

**Fig. 6:**
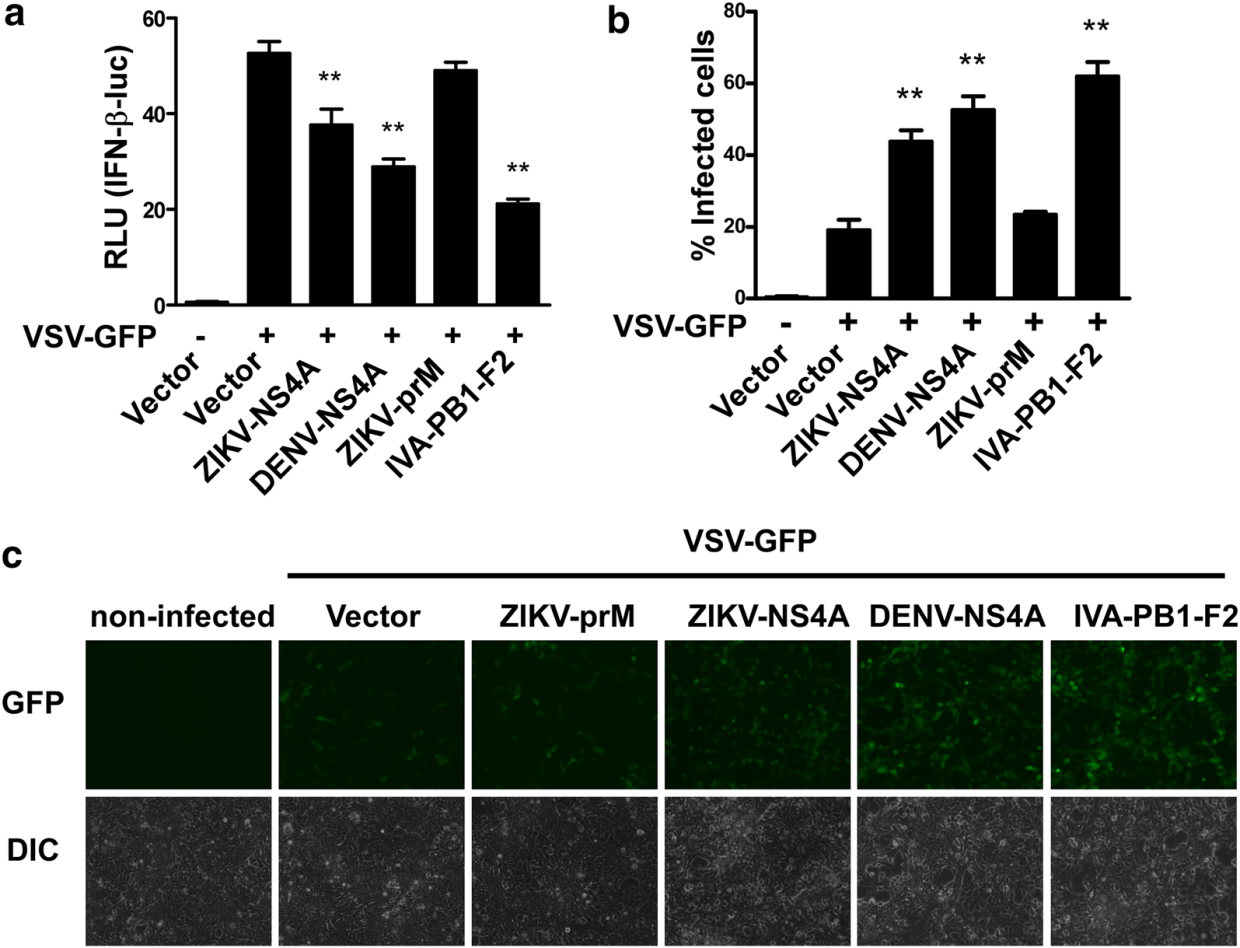
ZIKV NS4A blocks VSV-induced IFN production. **a** 293T cells transfected with IFN-β reporter together with ZIKV-NS4A, DENV-NS4A, ZIKV-prM and IVA-PB1-F2 (500 ng/well) were infected with VSV-GFP for 24 h, and cell lysates were then analyzed for reporter luciferase activity. The data are shown as the mean ± SD derived from three repeat experiments. **p < 0.01 (Student’s *t* test). **b**, **c** 293T cells were transfected with empty vector, and a ZIKV-NS4A-, DENV-NS4A-, ZIKV-prM- or IVA-PB1-F2-expressing plasmid. Twenty-four hours later, the cells were infected with SeV, and the supernatants were UV-treated and used to overlay 293T cells. Twenty-four hours later, the cells were infected with VSV-GFP for 24 h, followed by scoring of the number of GFP-positive cells by flow cytometry (**b**), and cell images were taken under a fluorescence microscope (**c**)

To further validate that ZIKV NS4A is an antagonist of IFN production, we employed a recombinant VSV-GFP virus system in our study, and our data displayed that ZIKV NS4A, DENV NS4A or IVA PB1-F2 remarkably reduced the IFN-β reporter activity upon VSV-GFP stimulation (Fig. 6a). Moreover, our results also revealed that the recombinant VSV-GFP virus in 293T cells expressing ZIKV NS4A, DENV NS4A or IVA PB1-F2 exhibited a high-degree replication (Fig. 6b, c), suggesting that the inhibitory effect of ZIKV NS4A on IFN production could occur in the scenario of actual viral infection.

## Discussion

The recent extensive epidemic out breaks of ZIKV infection and its dissemination to various regions of the world, as well as its link to severe complications, particularly microcephaly in the newborns, have created a serious threat to global health [1]. The rapidly emerging health crisis associated with ZIKV infection highlights a growing need to understand the mechanisms by which ZIKV accesses human hosts. Evidence for a crucial role of the type I IFN response in preventing infection lies in the fact that ZIKV has evolved a variety of strategies to subvert the host defense by counteracting early infection-triggered IFN induction or subsequent IFN-triggered signaling. In the present study, we demonstrated that by utilizing its NS4A protein, ZIKV could suppress IFN production through disrupting the RIG-I–MAVS signaling pathway. This finding broadens and deepens our knowledge of how ZIKV has developed diverse strategies to evade the action of IFN.

It has been suggested that flaviviruses have evolved complex mechanisms to evade host anti-viral mechanisms, including RLR signaling [7]. Our previous work revealed that NS4A proteins derived from various serotypes of DENV blocked type I IFN production through targeting MAVS-mediated signaling pathways [22]. Recently, Ma et al. reported that ZIKV NS4A in particular as a suppressor of the RLR pathway by interrupting RLR–MAVS interaction, preventing induction of type I IFNs and inhibiting ZIKV replication [25]. Toward understanding the counter-IFN capability of ZIKV in depth, our current work is featured in several aspects that are worth highlighting. Firstly, by employing surface plasmon resonance technology we here provide the first and direct evidence demonstrating that ZIKV NS4A directly binds MAVS protein and there by impair IFN production. Secondly, we have documented that ZIKV is a weak inducer of type I interferon in ZIKV-infected humans and in cultured human cell lines and primary cells as well. Lastly, while overexpression of ectopic NS4A was employed in our study, serial experiments in the context of actual ZIKV infection were performed, and clearly demonstrated that NS4A inhibited IFN induction through an NS4A-MAVS interaction.

At the molecular level, while antagonism of IFN-mediated signal transduction by ZIKV NS4A shares multiple similarities with that of DENV NS4A, our findings revealed that ZIKV NS4A bound MAVS through specifically interacting with both the CARD and TM domains of MAVS. As reported, the CARD-like domain of MAVS is responsible for interacting with RIG-I, and the TM domain-containing region of MAVS has been found to mediate oligomerization and interaction with other adaptor proteins, such as tumor necrosis factor receptor-associated factor 3 (TRAF3) and TRAF6 [16]. It remains possible that NS4A also disrupts MAVS oligomerization or complexes with other adaptor proteins, which is an issue worth further investigation. Interestingly, type III IFN has been shown to protect barrier cells of the human placenta called trophoblasts from ZIKV infection. Type III IFN utilizes different cell-surface receptors than type I IFN but utilizes the same MAVS signaling machinery to regulate its production [26]. Thus, it is highly likely that ZIKV can also evade type III IFN signaling through targeting MAVS by NS4A, which may contribute to the ability of ZIKV to cross the placenta during pregnancy and consequently cause neuronal disorders in the developing fetus. Additionally, it would be of great interest to further identify the amino acids of MAVS responsible for the interaction between NS4A and MAVS. Since there is no three-dimensional structural information for NS4A, it is in fact very difficult to perform molecular docking experiments and identify the high-affinity binding sites between two proteins. Ding et al. revealed residues critical for ZIKV NS2B3-mediated cleavage to R78 and G79 in the cytoplasmic loop of human STING, but not mouse STING [17]. This finding needs to be further verified as residues R78 and G79 are only weakly conserved in the murine ortholog of STING. Human MAVS shares 51.8% amino acid identity with mouse MAVS, and thus, it may be of interest to further investigate whether NS4A can disrupt the interaction between mouse MAVS and its usual binding partners. Moreover, it might be equally important to further map the amino acids of ZIKV NS4A involved in IFN antagonism, which should allow generation of mutant ZIKVs that are attenuated due to their inability to prevent IFN signaling.

It is currently not known what has triggered the surge of recent epidemics of ZIKV and the associated severe diseases [1]. Mechanistically, aberrant suppression of the antiviral property of IFNs might facilitate infection of ZIKV as well as viral spreading in human populations. ZIKV is largely a mosquito-borne virus and can also be transmitted through sexual contact, blood transfusion, organ transplantation, and potentially via urine or saliva as well. Moreover, ZIKV may be transmitted from pregnant women to fetuses by a transplacental route [1]. In consistence with such a dynamic transmission pattern, ZIKV exhibits a broad tropism and ability to persist in multiple types of tissues/organs and in body fluids, including the eyes, blood, testis and semen [1, 2, 27, 28]. It is also of note that immunocompromised human subjects or mice have increased susceptibility to ZIKV infection and are more vulnerable to developing severe disease than healthy individuals [8, 9]. Moreover, it has been well documented that nonstructural proteins of ZIKV play important roles in the life cycle of ZIKV and that several of them, including NS1, NS2B/3, NS4A, NS4B, and NS5, interact with innate immune molecules [1]. We therefore hypothesize that in addition to mediating the effects of putative entry receptors on host cells being a key ZIKV infection mechanism, the ability of ZIKV to defeat the host IFN system, possibly through expressing multiple viral factors that interfere with multiple steps and nodes along the IFN signaling cascade, may also represent a key contributor to its broad cellular tropism and intracellular persistence. Hence, collaborative actions among different ZIKV nonstructural proteins need to be better understood, and further investigation of how the effect of NS4A, as identified in our current study, cooperates with other counter-IFN effects of ZIKV, such as NS1, NS2B/3, NS4A, NS4B, and NS5, is warranted.

## Conclusions

In summary, the current findings revealed an innate immunity evasion by ZIKV and the possible underlying molecular mechanisms. We demonstrated that the ZIKV NS4A protein plays an important role in suppressing interferon production through binding MAVS and disrupting the RIG-I–MAVS interaction in host cells. The identified function of ZIKV NS4A as an IFN antagonist might enable it to play an important role in the pathogenesis and spread of ZIKV in humans. This work has not only found a viral protein that participates in immune evasion, but also unveiled weaknesses in host defenses, thereby providing fundamental knowledge for the future development of anti-Zika drugs or vaccines.

## Materials and methods

### Patient samples

ZIKV-infected patients were recruited to the study from Enping People’s Hospital (Guangdong, China). Three hospitalized individuals from a family (a 40-year-old father, an 8-year-old daughter and a 6-year-old son, or patients A, B, and C) were diagnosed with ZIKV infection at Enping People’s Hospital in 2016. Clinical and demographical characteristics of the study subjects were summarized in our group’s previous publication [23]. The control subjects were three healthy volunteers without ZIKV infection. All human subjects gave written, informed consent to participate in accordance with the Declaration of Helsinki before sample collection. The protocols and informed consent forms used in this study were reviewed and approved by the Scientific and Ethical Committee of Guangzhou Women and Children’s Medical Center.

### mRNA profiling

Peripheral blood was collected from three patients during the onset period and from three healthy donors by using anticoagulant vacuum blood collection tubes (BD Franklin Lakes, NJ), and whole blood cells were subsequently collected by centrifugation at 800×*g* for 20 min at room temperature. Total RNA was extracted from the collected blood cells with TRIzol reagent (Invitrogen, Carlsbad, CA) according to the manufacturer’s instructions. Host cell transcriptional profiling was performed using RNA deep sequencing by employing the Annoroad Gene Technology Co., Ltd. (Beijing, China). Briefly, library construction was performed following the manufacturer’s instructions provided by Illumina (San Diego, CA, USA). The prepared libraries were sequenced on an Illumina HiSeq 2500 PE150 platform and 150 bp paired-end reads were generated. The raw sequencing data were submitted to the NCBI Gene Expression Omnibus (GEO) database [29] with GEO series accession number GSE123835. TopHat was used to analyze the RNA-seq data, and HTSeq v0.6.0 [30] was adopted to generate the count matrix, with default parameters. Reads PerKilobase Million Mapped Reads (RPKM) [30] was then calculated to estimate the expression level of genes in each sample. DEGseq v1.18.0 [30, 31] was used for differential gene expression analysis between two samples with non-biological replicates. A *p*-value was assigned to each gene and adjusted by Benjamini and Hochberg’s approach for controlling the false discovery rate [18, 30]. Differentially expressed genes (DEGs) were defined as those displaying log_2_ (fold change) ≥ 1.5 and a *p* value < 0.05 relative to the control cohort. Heatmaps were produced with the Gplots package and Heatmap.2 programs in R [18, 30].

### Cell culture and virus

293T human embryonic kidney cells (the Cell Bank of the Chinese Academy of Sciences, Shanghai, China), HeLa human cervical adenocarcinoma cells (ATCC) and HFF-1 human foreskin fibroblasts cells (the Cell Bank of the Chinese Academy of Sciences, Shanghai, China) were cultured in DMEM (Invitrogen, Carlsbad, CA) containing 10% fetal bovine serum (FBS) (GIBCO, Carlsbad, CA), 2 mM l-glutamine, 100 μg/ml streptomycin and 100 units/ml penicillin (Invitrogen, Carlsbad, CA) at 37 °C under 5% CO_2_. SV-HUC-1 human uroepithelial cells (the Cell Bank of the Chinese Academy of Sciences, Shanghai, China) were cultured in F12K medium (GIBCO, Carlsbad, CA) containing 10% FBS at 37 °C under a 5% CO_2_. C6/36 *Aedes albopictus* cells (ATCC, CRL-1660) were maintained at 28°C with 5% CO_2_ in DMEM supplemented with 10% FBS. Primary monocytes were purified from peripheral blood mononuclear cells (PBMC) from health donor 2 by using Monocyte Isolation Kit II (Miltenyi Biotec GmbH, Bergisch Gladbach, Germany) according to the manufacturer’s protocol. The background of healthy donor 2 is summarized and compared in Additional file 1: Table S1. Zika virus strain ZG-01 (GenBank accession number KY379148.1) was isolated by our group in 2016 from the urine of an infected 40-year-old man (patient A) [23]. Sendai virus (SeV) was grown in 10-day-old embryonated chicken eggs and titrated by hemagglutination assay as previously described [22].

### Plasmids

The cDNA coding for each ZIKV protein, including prM, NS4A and NS4A-Flag (with a C-terminal Flag epitope tag), was PCR amplified from ZG-01 strain viral cDNA as a template and cloned into pFN11A (Promega, San Luis Obispo, CA) or pcDNA3.1 vectors. Plasmids including pcDNA3.1-DENV NS4A, -IVA PB1-F2, -MAVS, -RIG-I (N), pFN10A (ACT)-RIG-I, -MAVS, -TBK1, and-IKKε were constructed as previously described [22]. Truncated forms of MAVS (aa 1–77, 74–173 and 174–540) with N-terminal Flag epitope tags were amplified from the full-length template and cloned into the pcDNA3.1 vector. The pIFN-β-luc reporter plasmid was constructed by cloning a 125 bp fragment of the IFN-β promoter into the pGL3-Basic vector using the *Nhe*I and *Hin*dIII sites upstream of the luciferase reporter gene [8, 22]. All constructs were verified by DNA sequencing. All primers used for plasmid construction are listed in Additional file 3: Table S3.

### Luciferase reporter assays

293T cells were co-transfected in a 24-well plate (1 × 10^5^ cells per well) with 100 ng of IFN-β promoter reporter plasmid, pRL-TK (5 ng), the plasmid coding for RIG-I (100 ng) together with a plasmid expressing ZIKV NS4A, ZIKV prM, DENV NS4A or IVA PB1-F2. Empty pcDNA3 vector was used as a transfection control. Thirty-six hours after transfection, the total cell lysate was measured using the Dual-Luciferase Reporter Assay System kit (Promega, San Luis Obispo, CA). When using SeV or poly(I:C) (Sigma-Aldrich, St. Louis, MO) as a stimulus, 293T cells transfected with IFN-β reporter, pRL-TK, together with ZIKV NS4A, ZIKV prM, DENV NS4A or IVA PB1-F2, were infected with or without SeV at 100 HAU/ml for 16 h, or transfected with 20 μg/ml of poly(I:C) for 16 h, followed by analysis of cell lysates for luciferase activity.

### Mammalian two-hybrid assay

293T cells were transfected using Lipofectamine 2000 as transfection reagent (Invitrogen, Carlsbad, CA) as described previously [32]. One hundred nanograms of pFN11A (BIND) vector expressing GAL4-BD fused with each individual Zika virus protein, and plasmid pFN10A (ACT) expressing the transcriptional activation domain of the herpes simplex virus VP16 (VP16-AD) fused to RIG-I, MAVS, TBK1 or IKKε protein, were cotransfected with 250 ng of the pGL4.31 reporter plasmid. Empty pBIND and pACT plasmids were used as negative controls, and the pBIND-Id and pACT-MyoD plasmids were used as positive controls. The transfected cells were harvested for the dual-luciferase reporter assay according to the manufacturer’s instructions (Promega, San Luis Obispo, CA) at 48 h following transfection.

### Biacore analysis

Surface plasmon resonance (SPR) experiments were performed in a Biacore T100 device (Biacore Inc., Uppsala, Sweden) using research grade CM5 sensor chips (General Electric Company, GE) according to the protocol provided by the manufacturer. For SPR analysis, recombinant Zika virus NS4A protein was purified using a Ni–NTA affinity column (The QIA expressionist™, Qiagen, Chatsworth, CA) followed by Superdex 200 (GE) gel filtration chromatography, as previously described [22]. Blank control samples were prepared from blank vector expression system that contained only the vector plasmid without the NS4A expression cassette. Briefly, recombinant human MAVS protein (purchased from OriGene, TP308175) was immobilized on a CM5 sensor chip after activation with 1-ethyl-3-(dimethylaminopropyl) carbodiimide and *N*-hydroxysuccinimide. Different concentrations (0, 62.5, 125, 250, 500, 1000, and 2000 nM) of purified ZIKV NS4A or prM protein, or 100 microliters of blank control samples were injected at a flow rate of 30 μl/min for 3 min. Subsequently, data were collected for a 3-min association followed by a 20-min dissociation. The chip was regenerated by injecting 10 μl of 15 mM NaOH for 20 s. All procedures were run with HBS (HEPES buffered saline; GE) as a running buffer. Binding curves were determined using BIA evaluation 3.1 software and its equation for 1:1 Langmuir binding. The K_D_ was calculated as previously described [22].

### Western blotting analysis

Cells were harvested and lysed in sample buffer (50 mM Tris–HCl [pH 7.4), 1 mM PMSF, 10% glycerol, 6% SDS, 5% mercaptoethanol and 0.1% bromophenol blue), and the protein concentration was determined by BCA protein assay (Thermo Fisher Scientific, Rockford, IL). Briefly, protein bands separated by sodium dodecyl sulfate polyacrylamide gel electrophoresis (SDS-PAGE) were transferred to a PVDF membrane and then blocked with blocking buffer (5% non-fat milk in Tris-buffered saline). Specific protein bands were detected using the following primary antibodies: anti-ZIKV E (Gene Tex Inc, Alton Pkwy Irvine, CA), anti-ZIKV NS4A (Gene Tex Inc, Alton Pkwy Irvine, CA), anti-MAVS (Bethyl Laboratories, Montgomery, TX), anti-TRAF6, anti-HA, anti-Flag M2, anti-c-Myc, anti-β-actin (Sigma-Aldrich, St. Louis, MO), and anti-TBK1/NAK (Cell Signaling, Danvers, MA) antibodies. Further incubation with a horseradish peroxidase-conjugated secondary antibody and signals were detected by enhanced chemiluminescence using a commercial kit (Thermo Fisher Scientific, Rockford, IL) according to the manufacturer’s suggested protocol.

### Immunofluorescence assay

HeLa cells were grown on coverslips and transfected with the indicated plasmid. At 24 h after transfection, cells were washed once with PBS and fixed in 4% paraformaldehyde in PBS. Subsequently, cells were permeabilized with 0.2% Triton X-100 and treated for 30 min at room temperature with 10% BSA in PBS, followed by incubation with primary antibody for 1 h. Primary anti-Flag or anti-MAVS antibodies (Santa Cruz Biotechnology, Santa Cruz, CA) was used, followed by incubation with FITC- or Rhodamine-conjugated secondary antibodies (Jackson Immuno Research Laboratories, West Grove, PA), and 4-,6-diamidino-2-phenylindole (DAPI)was used to stain the nuclei. The coverslips were washed extensively and fixed on slides, and images were taken under an LSM800 confocal microscope (Carl Zeiss MicroImaging GmbH, Jena, Germany).

### Co-immunoprecipitation assay

293T cells were transfected with the indicated plasmids, and whole-cell lysates were prepared after transfection with protein lysis buffer containing 25 mM HEPES, 150 mM NaCl, 1 mM EDTA, 2% glycerol, 1% NP40, and a cocktail of protease and phosphatase inhibitors (Roche, Basel, Switzerland). Lysates were incubated with the indicated antibodies (anti-Flag M2, anti-Myc antibody anti-NS4A, anti-MAVS, or IgG antibody, Sigma-Aldrich, St. Louis, MO) at 4 °C overnight. Subsequently, lysates were incubated with Dynabeads protein A (Life Technologies, Grand Island, NY) according to the manufacturer’s suggested protocol. The precipitates were washed with wash buffer (20 mM HEPES, 150 mM NaCl, 1 mM EDTA, 1 mM EGTA, 2% glycerol and 0.1% NP40) for five times, resuspended in sample buffer, and assessed with Western blotting analysis.

### Quantitative real-time PCR (qRT-PCR)

Total RNA from cells was extracted with TRIzol reagent (Invitrogen, Carlsbad, CA) according to the manufacturer’s instruction. The RNA concentration was determined in a spectrophotometer at 260 nm, and 500 ng of RNA was reverse transcribed using random hexamer primers, and qPCR was carried out using FastStart Universal SYBR Green Master Mix (Roche, Basel, Switzerland). Expression levels for individual mRNAs were calculated and normalized based on their CT values using housekeeping genes (GAPDH) to normalize the data. Primers sets used for qPCR are shown in Additional file 3: Table S3.

### Quantification of IFN by ELISA

293T cells were transfected with the indicated plasmid for 24 h, followed by infection with or without SeV at 100 HAU/ml, or transfected with or without 20 μg/ml of poly(I:C) for 24 h. The supernatants were quantified for IFN-β protein using the Human Interferon-β ELISA Kit (PBL Assay Science, New Jersey, USA) according to the manufacturer’s instructions. Absorbance at 450 nm was determined on a Bio-Tek Synergy 2 microplate reader.

### Statistical analysis

The results are expressed as the mean ± standard deviation (SD). Statistical analyses were performed on triplicate experiments using a two-tailed Student’s t test.

## Additional files

**Additional file 1: Table S1.** The clinical characteristics of three individuals with ZIKV infection and the backgrounds of three healthy donors.

**Additional file 2: Table S2.** Functional categories of the 69 pre-defined antiviral ISGs in vivo.

**Additional file 3.** Additional figures and table.

## Data Availability

All data generated or analyzed during this study are included in this published article and its additional files.

## Abbreviations

ZIKV: Zika virus
IFN: interferon
JEV: Japanese encephalitis virus
DENV: dengue virus
YFV: yellow fever virus
WNV: West Nile virus
IVA: influenza
PAMPs: pathogen-associated molecular patterns
PRRs: pathogen recognition receptors
RLRs: RIG-I-like receptors
cGAS: cGMP-AMP synthase
CARD: caspase activation and recruitment domains
CL: CARD-like
PR: proline-rich
TM: transmembrane
FL: full-length
MAVS: mitochondrial antiviral signaling adaptor
MAMs: mitochondrion or mitochondria-associated membranes
DCs: dendritic cells
NS: nonstructural protein
DEGs: differentially expressed genes
FBS: fetal bovine serum
SeV: Sendai virus
SPR: surface plasmon resonance
SD: standard deviations
RNA-seq: RNA sequencing
