# Supplementary material for: Zika virus antagonizes interferon response in patients and disrupts RIG-I–MAVS interaction through its CARD-TM domains
Source: Cell Biosci. 2019 Jun 7;9:46. doi: 10.1186/s13578-019-0308-9 (PMC6555941; doi:10.1186/s13578-019-0308-9)
Supplement: Supplementary file 3 — Additional file 3. Additional figures and table. [file 13578_2019_308_MOESM3_ESM.doc]

**Additional Figure S1**


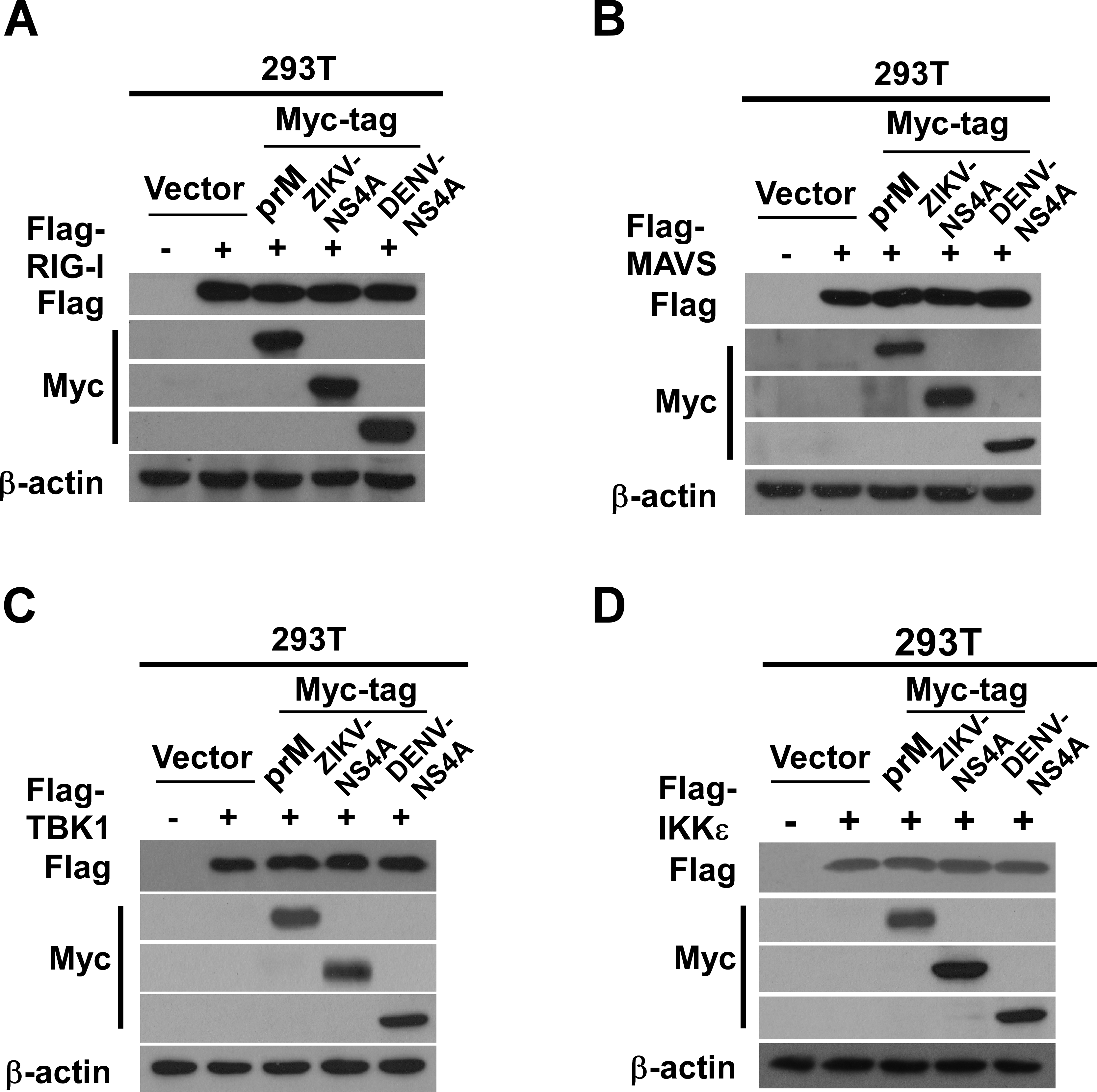


**Figure S1. Validation of protein expression of ZIKV NS4A and components of RLR signaling pathway used in the mammalian two-hybrid system.** 293T cells in 24-well plates were co-transfected with pGL4.31 vector, together with pFN10A(ACT) vector expressing Flag-tagged RIG-I (A), MAVS (B), TBK1 (C) or IKKε protein (D), and pFN11A(BIND) vector expressing a fusion protein of GAL4-BD and individual viral protein, including Myc-tagged ZIKV prM, ZIKV NS4A and DENV NS4A. At 48 h post-transfection, the whole cell lysates were harvested and analyzed by western blotting with indicated antibodies. The data shown are representative of three independent experiments.

**Additional Figure S2**


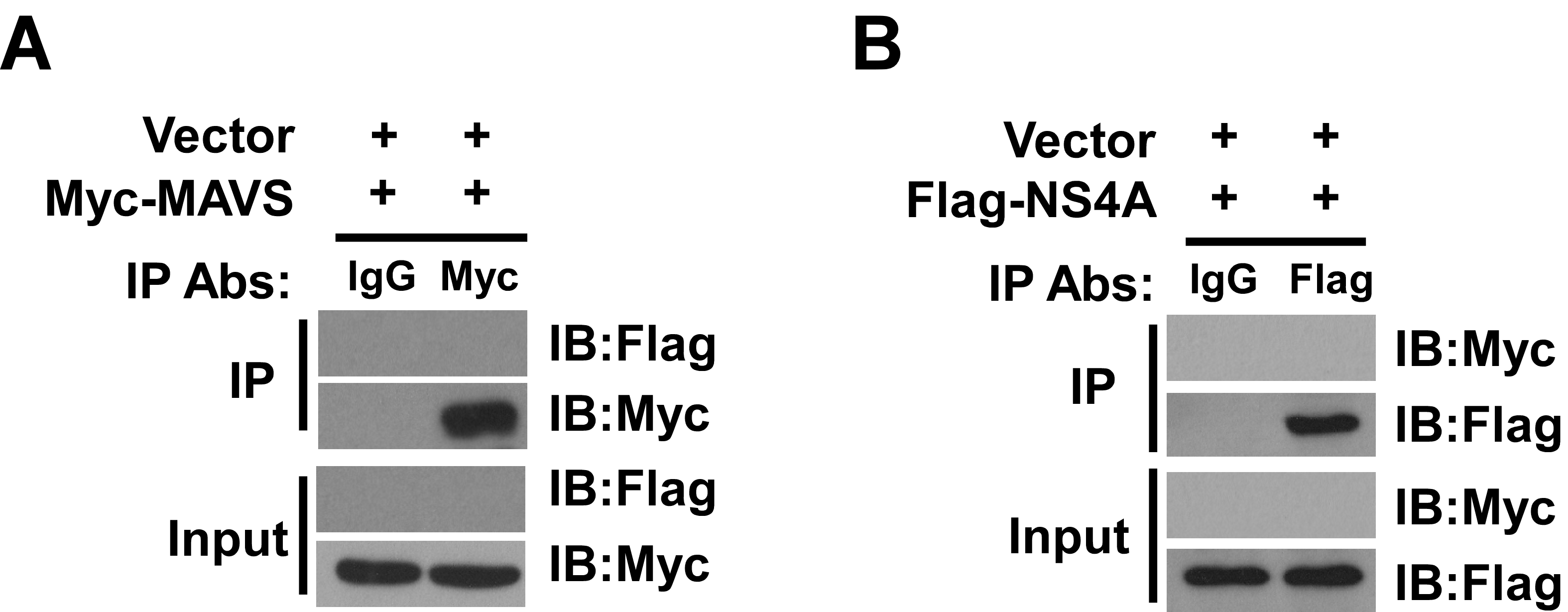


**Figure S2. (A)** 293T cells were co-transfected with Myc-tagged MAVS and pcDNA3.1 vector, and cell lysates were precipitated with anti-Myc antibody or control mouse IgG. **(B)** 293T cells were co-transfected with Flag-tagged NS4A and pcDNA3.1 vector, and cell lysates were precipitated with anti-Flag antibody or control mouse IgG. And immunocomplexes were analyzed with the indicated antibodies by western blotting. The data shown are representative of three independent experiments with similar results.

**Additional Figure S3**


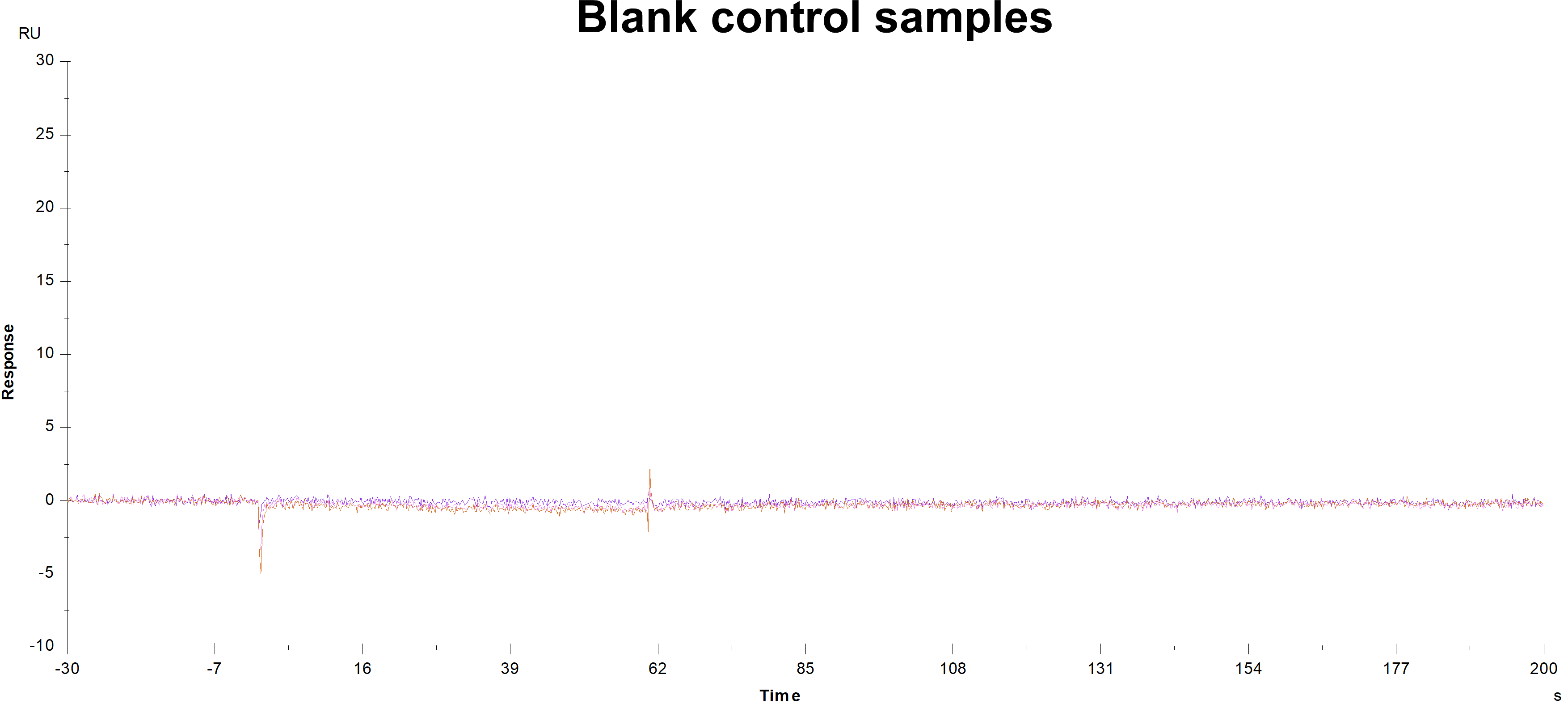


**Figure S3. SPR analysis of the interactions between MAVS and blank control samples.** Direct binding was measured by Biacore assays. MAVS was immobilized on a CM5 chip. Blank control samples were prepared from blank vector expression system that contained only the vector plasmid without the NS4A expression cassette. The analytes consisted of 100 microliters of blank control samples (an equal volume of recombinant Zika virus NS4A protein used in Figure 3F). The data shown are representative of three independent experiments with similar results.

**Additional Figure S4**


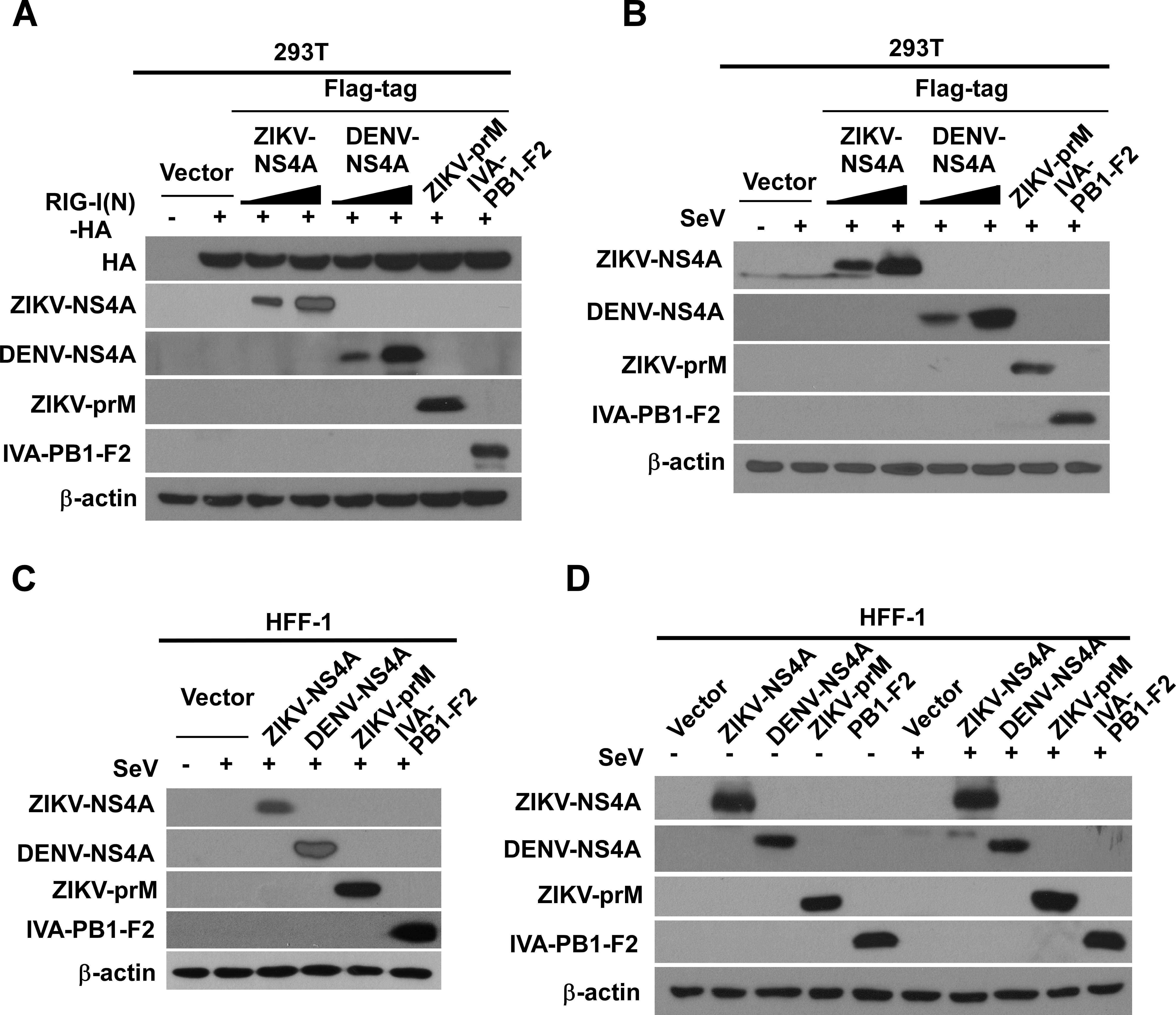


**Figure S4. Validation of protein expression of ZIKV NS4A, DENV NS4A, ZIKV prM or IVA PB1-F2 in cells to determine the quantity or activity of IFN-β.** The protein expression of ZIKV NS4A, DENV NS4A, ZIKV prM and IVA PB1-F2 used in the IFN-β luciferase reporter assay, ELISA and real time PCR analysis as shown in Figure 5 were validated by western blotting analysis. Data shown here are representative of three independent experiments with similar results.

**Additional Table S3. Primer sequences used in this work**

|  | Primers | Sequence (5’-3’) |
| --- | --- | --- |
| Myc-MAVS | Forward | CGGGGTACCGCCATGGAACAAAAACTTATTTCTGA  AGAAGATCTGCCGTTTGCTGAAGACAAG |
|  | Reverse | TGCTCTAGACTAGTGCAGACGCCGCC |
| Flag-MAVS (1-77 aa) | Forward | CGGGGTACCGCCATGGATTACAAGGATGACGACG  ATAAGCCGTTTGCTGAAGACAAG |
|  | Reverse | TGCTCTAGACTACCTCAGTGCCGCAATGAAGTAC |
| Flag-MAVS (78-173 aa) | Forward | CGGGGTACCGCCATGGATTACAAGGATGACGACG  ATAAGGGCTGTGAGCTAGTTGATC |
|  | Reverse | TGCTCTAGACTATGGATTCCTTGGGATGGCTCTG |
| Flag-MAVS (174-540 aa) | Forward | CGGGGTACCGCCATGGATTACAAGGATGACGACG  ATAAGGATGGTGGCCCCCTGGAG |
|  | Reverse | TGCTCTAGACTAGTGCAGACGCCGCC |
| Flag-MAVS (1-540 aa) | Forward | CGGGGTACCGCCATGGATTACAAGGATGACGACG  ATAAGCCGTTTGCTGAAGACAAG |
|  | Reverse | TGCTCTAGACTAGTGCAGACGCCGCC |
| RIG-I(N)-HA | Forward | CGGGGTACCGCCATGACCACCGAGCAGCGA |
|  | Reverse | TGCTCTAGACTAAGCGTAGTCTGGGACGTCGTATGGG  TATTTTTTAAGATGATGTTC |
| Myc-NS4A | Forward | CGGGGTACCGCCATGGAACAAAAACTTATTTCT  GAAGAAGATCTGGGAGCGGCTTTTGGAGTG |
| Flag-NS4A | Forward | CGGGGTACCGCCATGGATTACAAGGATGA  CGACGATAAGGGAGCGGCTTTTGGAGTG |
| His-NS4A | Forward | CGGGGTACCGCCATGCACCACCACCACCA  CCACGGAGCGGCTTTTGGAGTG |
|  | Reverse | TGCTCTAGACTAGGCGGTAATCAAGCCCAGA |
| Myc-prM | Forward | CGGGGTACCGCCATGGAACAAAAACTTATTTCTG  AAGAAGATCTGGCGGAGGTCACTAGACGTGG |
|  | Reverse | CGCGGATCCTCAGCTGTATGCCGGGGCAAT |
| pIFN-β-luc | Forward | CTAGCTAGCGTTTTAGAAACTACTAAAATGTAAATG |
|  | Reverse | CCCAAGCTTGAAAGGTTGCAGTTAGAATGTCC |
| pFN11A(BIND)-prM-Myc | Forward | CCGCGCGATCGCCATGGCGGAGGTCACTAGACGTGG |
|  | Reverse | AGTTGTTTAAACCTACAGATCTTCTTCAGAAATAAG  TTTTTGTTCGCTGTATGCCGGGGCAAT |
| pFN11A(BIND)-NS4A-Myc | Forward | CCGCGCGATCGCCATGGGAGCGGCTTTTGGAGTGAT |
|  | Reverse | AGTTGTTTAAACCTACAGATCTTCTTCAGAAATAAG  TTTTTGTTCGGCGGTAATCAAGCCCAGAA |
| pFN11A(BIND)-DV2-NS4A-myc | Forward | CCGCGCGATCGCCATGTCCCTGACCCTGAACCTAATCACA |
|  | Reverse | AGTTGTTTAAACTCACAGATCTTCTTCAGAAATAAGTTTTTGTTCTCTCTGCTTTTCTGGTTCTGGAATAAG |
| pFN10A(ACT)-TBK1-Flag | Forward | CCGCGCGATCGCCATGGATTACAAGGATGACGACGA  TAAGCAGAGCACTTCTAATCATCTGTG |
|  | Reverse | AGTTGTTTAAACCTAAAGACAGTCAACGTTGCG |
| pFN10A(ACT)-MAVS-Flag | Forward | CCGCGCGATCGATTACAAGGATGACGACGATAAGGCCA  TGCCGTTTGCTGAAGACAAG |
|  | Reverse | AGTTGTTTAAACCTAGTGCAGACGCCGCC |
| pFN10A(ACT)-RIG-I-Flag | Forward | CCGCGCGATCGATTACAAGGATGACGACGATAAGGC  CATGACCACCGAGCAGCGA |
|  | Reverse | AGTTGTTTAAACTCATTTGGACATTTCTGCTGGATC |
| pFN10A(ACT)-IKKε-Flag | Forward | CCGCGCGATCGATTACAAGGATGACGACGATAAGG  CCATGCAGAGCACAGCCAATTACC |
|  | Reverse | AGTTGTTTAAACTCAGACATCAGGAGGTGCTGGG |
| *IFNB* | Forward | CATTACCTGAAGGCCAAGGA |
|  | Reverse | CAATTGTCCAGTCCCAGAGG |
| *OAS1* | Forward | CCAAGCTCAAGAGCCTCATC |
|  | Reverse | GAGCTCCAGGGCATACTGAG |
| *IFITM1* | Forward | TCATCCTGTCACTGGTATTCGGCTC |
|  | Reverse | GTGGGTATAAACTGCTGTATCTAGGG |
| *GAPDH* | Forward | GACTCATGACCACAGTCCATGC |
|  | Reverse | AGAGGCAGGGATGATGTTCTG |
| SeV | Forward | AATAGGGACCCGCTCTGTCT |
|  | Reverse | TTCCACGCTCTCTTGGATCT |
